# Suppression of multiple myeloma by mitochondrial targeting

**DOI:** 10.1038/s41598-021-83829-2

**Published:** 2021-03-12

**Authors:** Yana Aisen, Moshe E. Gatt, Rachel Hertz, Elia Smeir, Jacob Bar-Tana

**Affiliations:** 1 Department of Human Nutrition and Metabolism, Hebrew University Medical School, 91120 Jerusalem, Israel; 2 Department of Hematology, Hadassah Medical Center, 91120 Jerusalem, Israel

**Keywords:** Cancer, Drug discovery, Diseases

## Abstract

Treatment of multiple myeloma (MM) aims at inducing cell apoptosis by surpassing the limited capacity of MM cells to cope with oxidative stress. MM cell survival may further be suppressed by limiting cellular cholesterol. Long-chain fatty acid analogs of the MEDICA series promote mitochondrial stress and inhibit cholesterol biosynthesis, thus prompting us to verify their efficacy and mode-of-action in suppressing MM cell survival, in comparison to bortezomib. MEDICA analog is shown here to effectively suppress survival of MM cells, and to inhibit growth of MM xenograft. Suppression of MM cell survival by MEDICA is accompanied by inhibition of the STAT3, MAPK and the mTORC1 transduction pathways due to mitochondrial oxidative stress. MEDICA-induced oxidative stress is abrogated by added exogenous cholesterol. Suppression of MM cell survival by bortezomib is similarly driven by bortezomib-induced oxidative stress, being abrogated by added cholesterol. In line with that, the time-to-best-response of MM patients to bortezomib-based treatment protocols is shown to be positively correlated with their plasma cholesterol level. MEDICA profile may indicate novel therapeutic potential in the management of MM.

## Introduction

Multiple myeloma (MM) is an incurable cancer of clonal mature B-lymphocytes^1^. It accounts for about 1% of cancer deaths and nearly 20% of deaths caused by hematological malignancies. It is driven by somatic oncogene mutations, being further promoted by the interplay between MM tumor cells and bone marrow stromal cells (BMSC), osteoclasts, osteoblasts, and extracellular matrix^1, 2^. These result in activating the IL-6/STAT3, PI3K/Akt/mTORC1 and MAPK transduction pathways that promote cell survival and suppress apoptosis^3^. The mutual connectivity of oncogenic drivers and their downstream transducers results in drug resistance, and in having to use a variety of drug combinations, including proteasome inhibitors (e.g., bortezomib), thalidomide analogues, cytotoxic drugs (e.g., melphalan, cyclophosphamide, or doxorubicin), steroids and radiotherapy^4^. Selective MM cytotoxicity by current medications is partly accounted for by drug-induced reactive oxygen species (ROS), that surpass the limited capacity of MM cells to cope with their inherent and drug-induced oxidative stress^5, 6^. In addition, previous reports have indicated efficacy of high-dose statins in suppressing MM cell survival^7–9^, due to inhibition of cholesterol biosynthesis^10^.

MEDICA analogs^11^ consist of long-chain, α,ω-dioic acids [HOOC-C(α’)-C(β’)-Q-C(β)-C(α)-COOH, where Q represents a core element], substituted in the αα’ (Mαα), ββ' (Mββ), and/or other optional core carbons. MEDICA analogs are not esterified into lipids, nor converted into ceramides, while the substitutions at the αα’ or ββ’ positions block their β-oxidation. MEDICA analogs are mostly excreted in bile as respective glucuronides. In analogy to natural long chain fatty acids^12, 13^, MEDICA analogs inhibit mitochondrial complex I of the electron transport chain^39^, resulting in ROS production, gating the mitochondrial permeability transition pore (mPTP)^14, 15^ and in activating AMPK^17^. Mitochondrial targeting by MEDICA results in suppressing mTORC1 activity^39^ and its downstream SREBP effector (Zatara et al., in preparation), resulting in inhibition of de novo cholesterol synthesis^16^. Inhibition of cholesterol synthesis by MEDICA analogs is further complemented by concomitant direct inhibition of the ATP-citrate lyase^16^. MEDICA proved preliminary safety in rats and dogs and in Phase I and IIa clinical studies (unpublished).

The combined activities of MEDICA in promoting mitochondrial oxidative stress and in inhibiting cholesterol biosynthesis prompted our interest in verifying their putative efficacy and mode-of-action in suppressing MM cell survival, in comparison to bortezomib. MEDICA analog and bortezomib appear to converge onto shared mode-of-action.

## Methods

### Animals

C.B-17/IsrHsd-SCID-bg 6–7-week-old female mice were inoculated subcutaneously with 10^7^ RPMI8226 cells in 50 μl RPMI1640 and 50 μl Matrigel (BD Biosciences). In line with our previous in vivo studies using MEDICA^17, 23^, experimental mice were fed a standard laboratory diet containing 40 mg MEDICA/kg body weight/day. Control mice were fed standard laboratory diet. Tumor width (W) and length (L) were measured every other day using caliper, and tumor volume was calculated by the formula 4π/3(((W + L)/4)^3^). Mice were sacrificed when approaching a tumor volume of 2 cm^3^. Upon sacrifice, tumors were removed, weighed and subjected to immunohistochemistry as indicated. The study was carried out in compliance with the ARRIVE guidelines (https://arriveguidelines.org).

### Cell lines

MM human cell lines U266, RPMI8226, NCl H929, JJN3, CAG, KMS11 and 5T33 murine cell line were cultured at 37 °C in humidified atmosphere containing 5% CO2 in RPMI1640 medium, supplemented with 10% Fetal Calf Serum (FCS), 2 mM l-Glutamine, 100U/ml penicillin and 0.1 mg/ml streptomycin (Biological Industries, Israel). Where stated, cells were cultured in the presence of added MEDICA analog [HOOC-C(CH_3_)_2_-(CH_2_)_12_-C(CH_3_)_2_-COOH] or bortezomib as indicated. Where stated, cells were cultured in the presence of added Tiron, EUK207, methylene blue, water-soluble cholesterol (Sigma Aldrich) or IL-6 as indicated. Bone marrow stromal cells (BMSC) were isolated and cultured as described^18^. Cell mitochondria were prepared by differential centrifugation^15^.

### MM primary cells

Patients’ MM CD138 + cells were selected by CD138 microbeads (Milteny Biotech) and were cultured in 20% FCS. Sampling of MM patient blood was approved by the Hadassah Medical Center IRB.

### Cell viability, cell cycle and apoptosis

Cell viability was determined by 3-(4,5-dimethylthiazol-2-yl)-2,5 diphenyl tetrasodium bromide (MTT) absorbance (Sigma Aldrich). For cell cycle analysis **c**ells (2 × 10^6^ cells/ml) were serum-starved overnight, cultured for 24 h in RPMI-1640/10% FCS medium with additions as indicated, fixated in ethanol, stained with propidium iodide (PI), and subjected to FACScan/CellQuest analysis (BD Biosciences). For apoptosis analysis cells were cultured in RPMI-1640/10% FCS medium with additions as indicated, stained by Annexin V-FITC (BD Pharmingen) and PI, and subjected to FACScan/CellQuest analysis (BD Biosciences).

### Immunohistochemistry

Formaldehyde-fixed paraffin-embedded sections were stained with antibodies and dyes as indicated and quantified by computerized microscopy (Ariol).

### Western blotting

Cells were lysed in lysis buffer (50 mM Tris–HCl (pH 8), 1% Triton, 1 mM EGTA, 1 mM EDTA, 150 mM NaCl, 5 mM NaPPi, 50 mM NaF, 1 mM PMSF, protease inhibitor cocktail (Sigma Aldrich), 0.2 mM Na-vanadate and 40 nM bpVphen (Calbiochem)). Protein concentration was determined by BCA (Thermo Scientific). Protein lysates were subjected to SDS-PAGE, transferred onto cellulose nitrate membranes (Schleicher & Schuell, Dassel, Germany) and probed with the indicated first antibody, followed by horseradish peroxidase-labeled second antibody. Bands were detected by ECL. The intensity of individual bands was analyzed by densitometry using TINA 2.10 software.

### RT-PCR

Total RNA of MM cells was prepared using the TRI reagent (Sigma Aldrich). First strand cDNA used as template was synthesized by reverse transcription using oligo(dT) or random hexamers mix as primer and the Reverse-iTMAX First Strand Kit (ABgene). Transcripts were quantified by real-time PCR (Rotor Gene RG-3000A) using SYBER green MasterMix (Absolute Syber Green ROX Mix, ABgene). Primer sequences were as follows: human Cyclin D1: forward (5-GTGCTGCGAAGTGGAAACC-3) and reverse (5-ATCCAGGTGGCGACGATCT-3); human C-Myc: forward (5-AGGCGAACACACAACGTCTT-3) and reverse (5-TTGGACGGACAGGATGTATGC-3); β-actin: forward (5-ATAGCACAGCCTGGATAGCAACGTAC-3) and reverse (5-CACCTTCTACAATGAGCTGCGTGTC-3); GAPDH: forward (5-GTTGCTGTAGCCAAATTCGTTG-3) and reverse (5-ACCCACTCCTCCACCTTTGA-3).

### ROS production

ROS production was determined by 2,7 dichlorofluoresceine diacetate (DCFDA) (5 μM) added to respective cell cultures for the last 15 min of incubation. Cells were washed once with PBS and analyzed by FACScan. Glutathione (GSH)/Glutathione disulfide (GSSG) ratio was determined by the GSH/GSSG-Glo assay kit (Promega).

### Mitochondrial superoxide production

Cells were cultured as indicated followed by adding 3.3 µg/ml MitoSOX (in DMSO) for the last 30 min. Cells were washed once with PBS and analyzed by FACScan.

### Mitochondrial membrane potential

Tetramethylrhodamine methylester perchlorate (TMRM, Molecular Probes) was added to the cell culture (25 nM final concentration) for the last 1 h of incubation. Cells were washed once with PBS and analyzed by FACScan.

### Mitochondrial cholesterol

Cells were incubated with water soluble cholesterol (Sigma Aldrich) as indicated. Mitochondria were isolated as previously described^15^. Mitochondrial cholesterol was determined by Amplex Red (Molecular Probes).

### Reagents

Anti-PARP (#9542), anti-Bcl-xL (#2762), anti-Bcl-2(#2876), anti-GAPDH (#2118), anti-cyclin D1 (#2922), anti-phospho-Rb(S807/811) (#9308), anti-Rb (#9309), anti-cleaved caspase 3 (#9661), anti-phospho-p70 S6 kinase(Thr389) (#9205), anti-S6 (#2217) and anti-phospho-S6(Ser240/244) (#5364) antibodies were from Cell Signaling Technology. Anti-phospho-STAT3(Y705) (sc-8059), anti-STAT3 (sc-7179), anti-phospho-Erk(T204) and anti-Mcl-1(sc-819) antibodies were from Santa Cruz Biotechnology. Anti-Erk (#06-182) and anti-gp130 (#06-291) antibodies were from Upstate. Anti-α-tubulin (T5168) antibody, water-soluble cholesterol (C4591) and Tyron were from Sigma Aldrich. Anti-Ki67 (#RM-9106-R7) antibody was from Thermo Scientific. Horseradish peroxidase-labeled secondary antibodies were from Jackson ImmunoResearch Laboratories. Secondary HRP-Polymer MACH3 (#M3RS31) antibody was from BioCare Medical. Secondary ImmPRESS (#MP-7401) antibody was from Vector Laboratories. Amplex Red Cholesterol assay kit (A12216) was from Molecular Probes. Recombinant IL-6 was from Pepro Tech. EUK207 was from Shirichai Orian, UCLA. MEDICA analog [HOOC-C(CH_3_)_2_-(CH_2_)_12_-C(CH_3_)_2_-COOH] was synthesized as previously described^11^. Bortezomib was from Janssen Pharmaceutica.

### Clinical data and analysis

Clinical data of newly diagnosed MM patients at the Hadassah Medical Center during January 2010 to June 2015 were retrospectively collected from patient files. Eligible patients had cholesterol levels prior to treatment initiation, and were treated uniformly with the same bortezomib, cyclophosphamide and dexamethasone (VCD) induction therapy^37^. Clinical data included baseline patient and disease characteristics, therapy and response rates. Response assessment was according to the consensus statement criteria of the myeloma working group^38^. Collection of patient data was approved by the Institutional Review Board of the Hadassah Medical Center.

### Statistics

All in-vitro experiments were performed at least in triplicate and repeated thrice. Statistical significance of differences was analyzed by unpaired t-test (P < 0.05) with Welch correction. Clinical data were analyzed by Microsoft excel data analysis for baseline statistics, and Graphpad prism software (version 5.03) for correlations and response analysis.

### Ethics

Animal care and experimental procedures were in accordance with the accredited animal ethics committee of the Hebrew University. Retrospective clinical data collection has been approved by the Institutional Review Board (IRB) of the Hadassah Medical Center. Being a retrospective study, no informed consent was required by the IRB.

## Results

### MEDICA suppresses MM tumor growth

**Figure 1:**
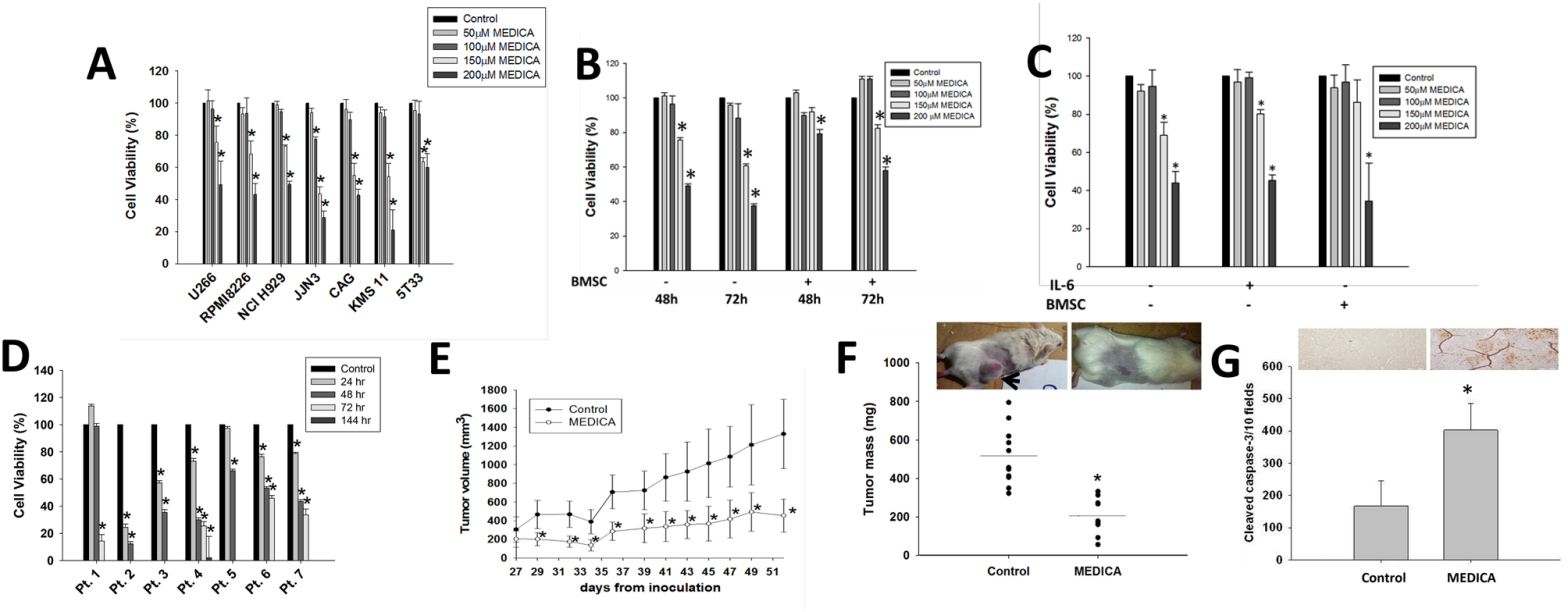
Suppression of MM cell proliferation by MEDICA. **(A)** Cell viability of MM cells cultured for 48 h with MEDICA as indicated. Mean ± SD of 5–8 replicates of 3–5 independent experiments. *Significant as compared with nontreated (p < 0.05). (**B)** Cell viability of U266 MM cells cultured with MEDICA in the presence or absence of BMSC as indicated. Mean ± SD of 5–8 replicates of 3 independent experiments. *Significant as compared with nontreated (p < 0.05). (**C)** Cell viability of RPMI8226 MM cells cultured for 48 h with MEDICA in the presence of BMSC and 10 ng/ml IL-6 as indicated. Mean ± SD of 5–8 replicates of 3 independent experiments. *Significant as compared with nontreated (p < 0.05). (**D)** CD138 + cells of MM patients were cultured for 24–144 h with 600 μM MEDICA as indicated. Mean ± SD of triplicates. *Significant as compared with nontreated (p < 0.05). (**E–G)** Mice were xenografted subcutaneously with RPMI8226 MM cells. Tumor volume **(E)**, tumor mass (**F**, inset—representative tumor image) and cleaved caspase 3 (**G**, Inset—representative histogram). Mean ± SD (n = 9–11). *Significant as compared with nontreated (p < 0.01).

Due to the molecular heterogeneity of MM cells, the effect of MEDICA analog [HOOC-C(CH_3_)_2_-(CH_2_)_12_-C(CH_3_)_2_-COOH] on MM cell viability was verified in a variety of human (U266, RPMI8226, NCIH929, JJN3, CAG, KMS11) and murine (5T33) MM cell lines (Fig. 1A). U266 and JJN3 MM cells express constitutive IL-6/STAT3. RPMI8226 and NCI H929 MM cells (non-constitutive for IL6/STAT3) express constitutive MAPK due to K-Ras mutations. CAG cells transplanted in SCID mice induce a clinical course of human MM. KMS11 cells represent late stage differentiated B-cells. MEDICA treatment resulted in a dose-dependent decrease in cell viability across all cell lines tested, with IC_50_ of 150 to 200 μM (50–70 μg/ml) (Fig. 1A). Due to the high binding affinity of MEDICA analogs to serum albumin (higher than 99%, independently of MEDICA concentrations in the range of 0–0.9 mM (Advinus study N079)), the µM concentrations of MEDICA in the culture medium in the presence of 10% FCS reflect nM concentrations of the free MEDICA acid. RPMI8226 and U266 MM cell lines have previously been reported to be sensitive and resistant to statins, respectively^19^, and were therefore further selected for verifying MEDICA mode-of-action in suppressing MM cell survival. MEDICA inhibited the growth of U266 and RPMI8226 MM cells co-cultured with bone marrow stromal cells (BMSC), as well as the growth of RPMI8226 cells in the absence or presence of added IL-6 (Fig. 1B,C). Higher concentrations of MEDICA or longer exposures were required for suppressing MM cell viability of MM/BMSC co-cultures. MEDICA showed similar efficacy in suppressing the growth of primary CD138^+^ MM cells (Fig. 1D). The higher MEDICA concentrations used here reflect the high FCS concentration (20%) required for maintaining growth of MM primary cells.

Suppression of MM tumor growth by MEDICA has been further verified in-vivo in SCID-bg mice transplanted subcutaneously with RPMI8226 cells, and treated with MEDICA mixed in diet (40 mg/Kg BW/ day). The oral dose used results in plasma steady state concentrations of ~ 50 μg/ml, namely in the range used in treating cultured MM cells. MEDICA treatment resulted in suppressing tumor growth (Fig. 1E) and tumor mass (Fig. 1F), and in inducing apoptosis as verified by cleaved caspase-3 expression (Fig. 1G). The Ki67 proliferation marker was not affected by treatment (not shown).

Combination therapy is currently the basis of MM treatment^1^. Compared to single agent treatment, doxorubicin or cyclophosphamide had a significant additive effect in combination with MEDICA, whereas combination of MEDICA with dexamethasone or bortezomib had neither additive nor antagonistic effects (Suppl Fig. 1).

### MEDICA induces MM cell cycle arrest and apoptosis

**Figure 2:**
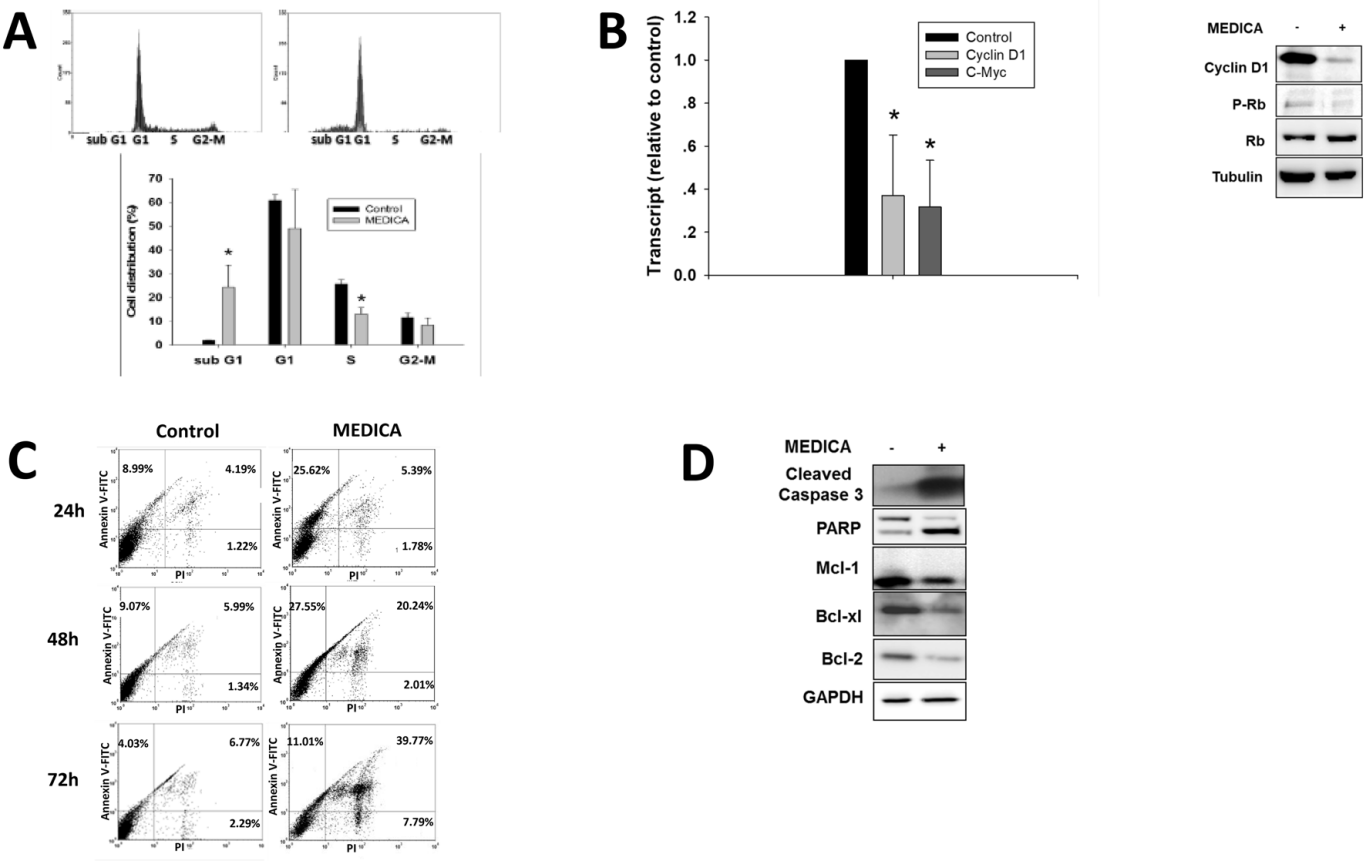
MM cell cycle arrest and apoptosis by MEDICA.** (A)** U266 MM cells were treated for 24 h with 200 μM MEDICA, stained with PI and subjected to FACS analysis. Mean ± SD of 3 independent experiments. *Significant as compared with nontreated (p < 0.05). Inset (upper frame)-representative histogram. (**B)** U266 MM cells were treated for 24 h with 200 μM MEDICA. Left-CD1 and C-Myc transcripts. Mean ± SD of 3 independent experiments. *Significant as compared with nontreated (p < 0.05). Right—representative blots. (**C)** U266 MM cells were treated for 24, 48 or 72 h with 200 μM MEDICA as indicated, stained with Annexin V-FITC/PI, and subjected to FACS analysis. Representative histograms. (**D)** U266 MM cells were treated for 24 h with 200 μM MEDICA. Representative blots.

MEDICA treatment of U266 cells resulted in decrease in S-phase cells (Fig. 2A), 70% decrease in Cyclin D1 (CD1) and C-Myc transcripts, and in CD1 and phospho-Rb proteins (Fig. 2B), implying cell cycle arrest. Growth arrest was further accompanied by apoptosis. Apoptosis was evident by 30-fold increase in sub-G1 cells (Fig. 2A) with increase in Annexin V-FITC positive cells (Fig. 2C). Apoptosis of U266 MM cells was accompanied by increase in PARP and cleaved caspase-3, with decrease in the anti-apoptotic Bcl-2, Bcl-xl, and Mcl-1 proteins (Fig. 2D). Similar results were observed in RPMI8226 (Suppl Fig. 2A,B) and 5T33 (Suppl Fig. 2C) MM cells.

### MEDICA inhibits the IL-6/STAT3, MAPK and mTORC1 transduction pathways

**Figure 3:**
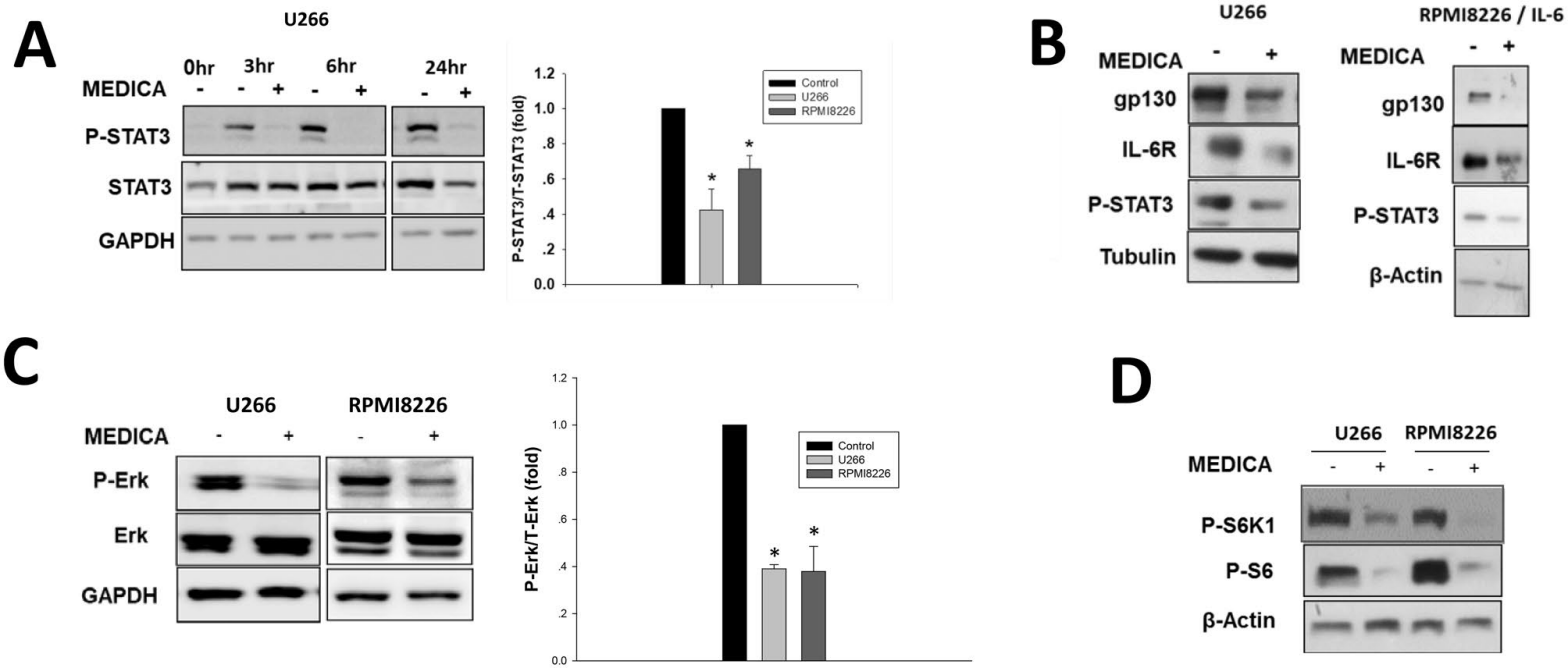
Suppression of transduction pathways by MEDICA. **(A,B**) U266 MM cells were treated with 200 μM MEDICA as indicated. RPMI8226 MM cells were treated for 24 h with 200 μM MEDICA and 20 ng/ml IL-6 added for the last 30 min as indicated. (**A)** Left—representative blots. Right—24 h phospho-STAT3/STAT3 ratio. Mean ± SD of 3 independent experiments. *Significant as compared with nontreated (p < 0.05). (**B**) Representative blots. (**C**,**D)** U266 and RPMI8226 MM cells were treated for 24 h with 200 μM MEDICA. (**C)** Left—representative blots. Right—24 h phospho-Erk/Erk ratio. Mean ± SD of 3 independent experiments. *Significant as compared with nontreated (p < 0.05). (**D**) Representative blots.

MEDICA treatment resulted in robust suppression of STAT3(Tyr705) phosphorylation in U266 (IL-6/STAT3 constitutive^20^) and RPMI8226 (MAPK constitutive) MM cells (Fig. 3A,B). Inhibition of STAT3(Tyr705) phosphorylation was already evident within the first 3 h of treatment, and was accompanied by decrease in STAT3 expression, due to the positive feedback of nuclear P-STAT3 in activating its own transcription^21^. Inhibition of STAT3(Tyr705) phosphorylation was accompanied by decrease in gp130 and IL-6Rβ cellular content (Fig. 3B), indicating that suppression of the IL-6/STAT3 transduction by MEDICA may partly be accounted for by abrogating the IL-6 receptor ligation. Of note, MEDICA inhibited the proliferation of U266 cells in which STAT3 was knocked out (not shown), implying additional MM oncogenic drivers beyond STAT3 that are suppressed by MEDICA. In line with that, MEDICA treatment resulted in suppressing Erk(Thr204) phosphorylation in U266 and RPMI8226 cells (Fig. 3C). In addition, MEDICA treatment resulted in suppressing mTORC1 activity as verified by its phospho-S6K1(Thr389) and phospho-S6(Ser240/244) downstream substrates (Fig. 3D).

### Mitochondrial ROS production by MEDICA and its abrogation by mitochondrial cholesterol

**Figure 4:**
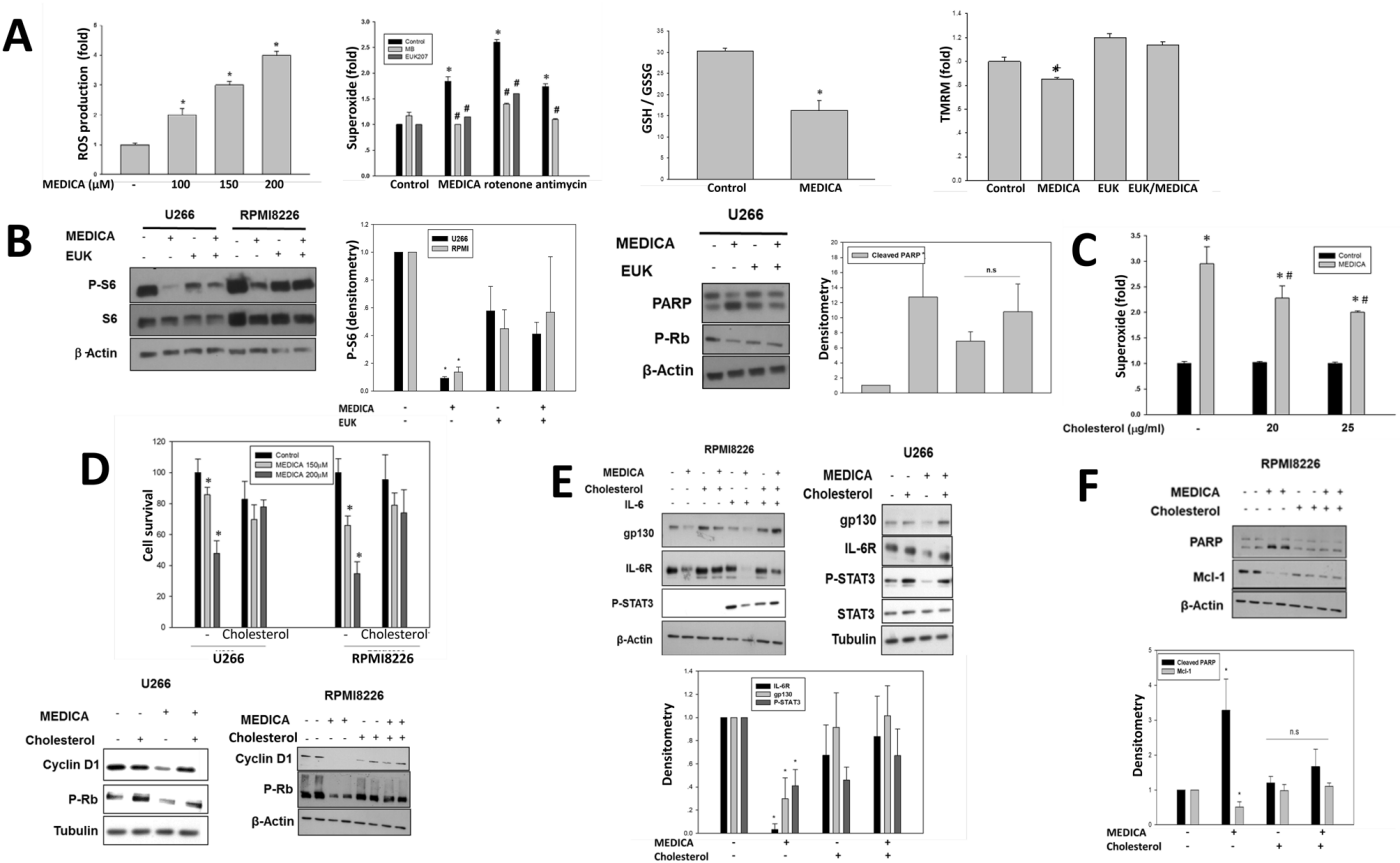
MEDICA-induced ROS production. (**A**) U266 MM cells were treated with 200 μM MEDICA, 2 μM rotenone, 2 μM antimycin or 20 μM EUK207 as indicated. Left to right—ROS production (5 h treatment); Mitochondrial superoxide production (5 h); GSH/GSSG ratio (24 h); Mitochondrial membrane potential (TMRM) (24 h). *Significant as compared with nontreated (p < 0.05). ^#^Significant as compared with MEDICA-rotenone- or antimycin-treated (p < 0.05). (**B**) U266 and RPMI8226 MM cells were treated for 24 h with 200 μM MEDICA and 20 μM EUK207 as indicated. Left—representative blots. Densitometry analysis of U266 blots (P-S6/actin) and RPMI8226 blots (P-S6/S6). Mean ± SD. *Significant as compared with nontreated (p < 0.05). Right—representative blots. Densitometry analysis of cleaved PARP. Mean ± SD. *Significant as compared with nontreated (p < 0.05). *n.s*. non-significant. (**C)** Superoxide production in U266 MM cells pre-treated with cholesterol for 20 h as indicated, rinsed and resuspended for 30 min in RPMI medium supplemented with 30 μM MEDICA. *Significant as compared with nontreated (p < 0.05). ^#^Significant as compared with MEDICA-treated in the absence of added cholesterol (p < 0.05). (**D)** U266 and RPMI8226 MM cells were treated with cholesterol and MEDICA as indicated. Upper—cell growth (20 μg/ml cholesterol, 72 h). *Significant as compared with nontreated (p < 0.05). Lower—representative blots (200 μM MEDICA, 30 μg/ml cholesterol, 24 h). (**E) **U266 MM cells were treated for 24 h with 20 μM MEDICA and 35 μg/ml cholesterol as indicated. RPMI8226 MM cells were treated for 24 h with 200 μM MEDICA, 35 μg/ml cholesterol and 20 ng/ml IL-6 added for the last 30 min as indicated. Representative blots. Densitometry analysis of RPMI8226 blots (IL-6R/actin, gp130/actin, P-STAT3(Tyr705)/actin). Mean ± SD. *Significant as compared with nontreated (p < 0.05). (**F)** RPMI8226 MM cells were treated for 24 h with 200 μM MEDICA and 35 μg/ml cholesterol as indicated. Densitometry analysis (cleaved PARP/actin, mcl-1/actin). Mean ± SD. *Significant as compared with nontreated (p < 0.05). *n.s.* non-significant.

MEDICA has recently been reported to inhibit mitochondrial complex I^39^, resulting in mitochondrial oxidative stress. MEDICA-induced mitochondrial superoxide and reactive oxygen species (ROS) production was studied in U266 MM cells by increase in cellular Mitosox and DCFDA fluorescence, with concomitant decrease in GSH/GSSG ratio (Fig. 4A). Increase in MEDICA-induced ROS was accompanied by mitochondrial depolarization as verified by decrease in TMRM (Fig. 4A). Mitochondrial superoxide production was similar to that induced by rotenone or antimycin. Mitochondrial depolarization by MEDICA was abrogated by the synthetic SOD/catalase EUK207 (Fig. 4A), resulting in abrogating MEDICA effects in activating PARP and in suppressing phospho-Rb and mTORC1 (Fig. 4B). Surprisingly, MEDICA effects were partially abrogated by increasing mitochondrial cholesterol content by added exogenous cholesterol. Thus, adding cholesterol to U266 MM cells for 24 h resulted in 2.5-fold increase in mitochondrial cholesterol content (3.0 and 7.0 µg cholesterol/mg mitochondrial protein in the absence and presence of added 25 µg cholesterol/ml, respectively). Added cholesterol resulted in partially abrogating MEDICA-induced mitochondrial superoxide production (Fig. 4C), growth inhibition (growth, CD1, phospho-Rb) (Fig. 4D), IL-6/STAT3 inhibition (phospho-STAT3, gp130, IL-6R) (Fig. 4E), and apoptosis (PARP, mcl-1) (Fig. 4F). Of note, abrogation of MEDICA effects by cholesterol was specific. Thus, PPARα activation by MEDICA^22^ was maintained in the presence of added cholesterol (not shown). Also, MEDICA activity in suppressing MM cell survival was not accounted for by lipid raft disruption as verified by plasma membrane caveolin-1 and GM1 content (not shown).

### Suppression of MM cell survival by bortezomib is mediated by mitochondrial ROS production and is partially abrogated by increase in mitochondrial cholesterol

**Figure 5:**
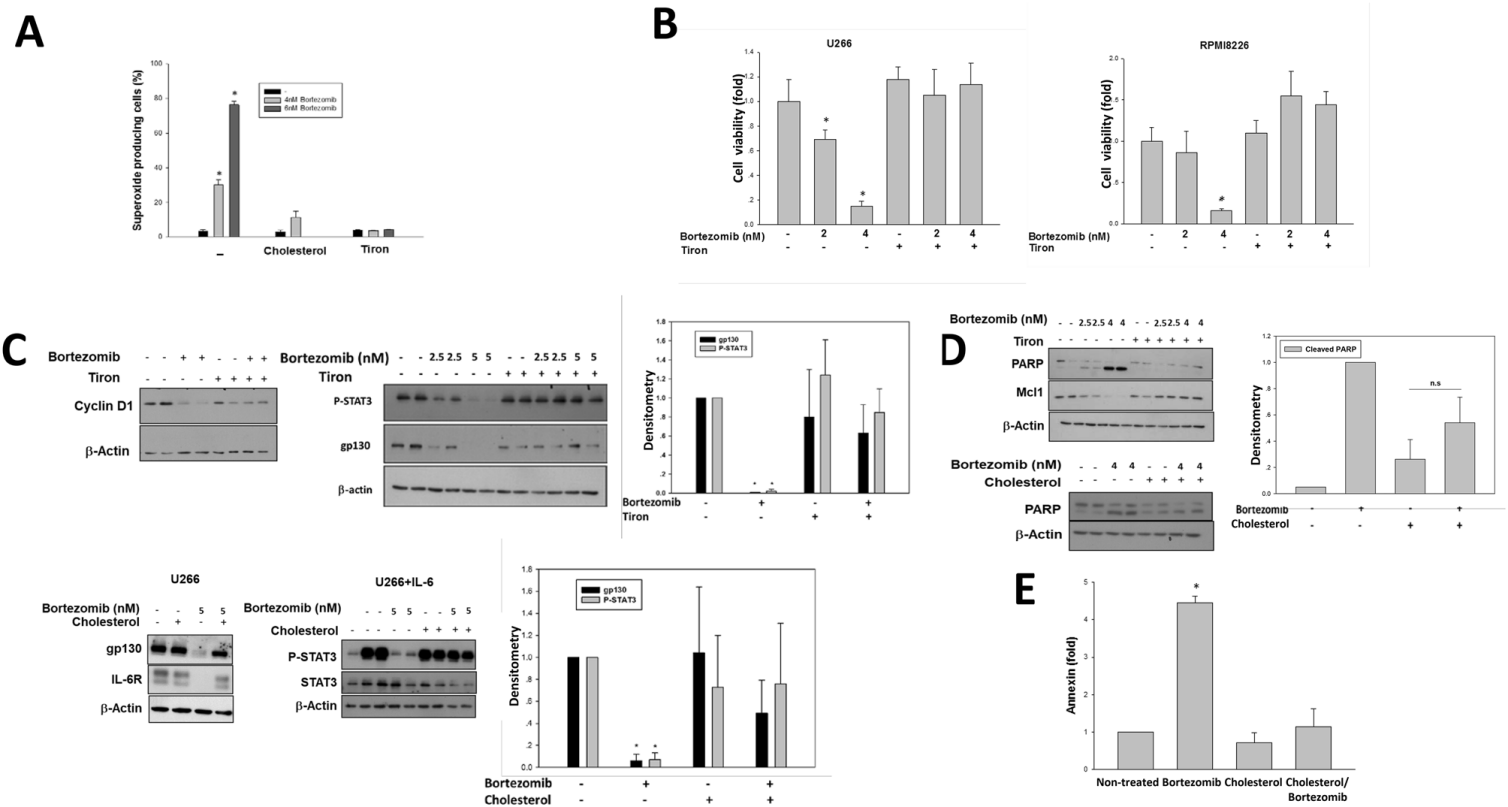
Bortezomib-induced ROS production. **(A)** U266 MM cells were treated for 24 h with 15 μg/ml cholesterol, 0.25 mM Tiron and bortezomib as indicated. Percentage of cells with high mitochondrial superoxide production. Mean ± SD of 3 independent experiments. *Significant as compared with nontreated (p < 0.05). (**B)** Viability of U266 and RPMI8226 MM cells cultured for 48 h with bortezomib and 0.25 mM Tiron as indicated. *Significant as compared with nontreated (p < 0.05). (**C)** U266 MM cells were cultured for 24 h with 5 nM bortezomib, 0.25 mM Tiron, 30 μg/ml cholesterol and 20 ng/ml IL-6 added for the last 30 min as indicated. Representative blots. Densitometry analysis (gp130/actin, P-STAT3(Tyr705)/actin). Mean ± SD. *Significant as compared with nontreated (p < 0.05). (**D)** U266 MM cells were treated for 24 h with 30 μg/ml cholesterol, 0.25 mM Tiron and bortezomib as indicated. Representative blots. Densitometry analysis (cleaved PAR/actin). Mean ± SD. *Significant as compared with nontreated (p < 0.05). *n.s.* non-significant. (**E)** U266 MM cells were treated for 24 h with 4 nM bortezomib and 20 μg/ml cholesterol as indicated. Mean ± SD of 3 independent experiments. *Significant as compared with nontreated (p < 0.05).

Combination of MEDICA with bortezomib had no additive effect in suppressing MM cell proliferation (Suppl Fig. 1D), implying possible shared target(s). Indeed, similarly to MEDICA, and in line with previous reports^5, 6^, treatment of U266 MM cells with bortezomib resulted in mitochondrial superoxide production, being abrogated by added Tiron (ROS scavenger) or cholesterol (Fig. 5A). Added Tiron or cholesterol further resulted in abrogating growth inhibition (Fig. 5B), CD1 and IL-6/STAT3 inhibition (phospho-STAT3, gp130, IL-6R) (Fig. 5C) and apoptosis (PARP, mcl-1, annexin) (Figs. 5D,E) by bortezomib.

### LDL-cholesterol and response to bortezomib in MM patients

**Figure 6:**
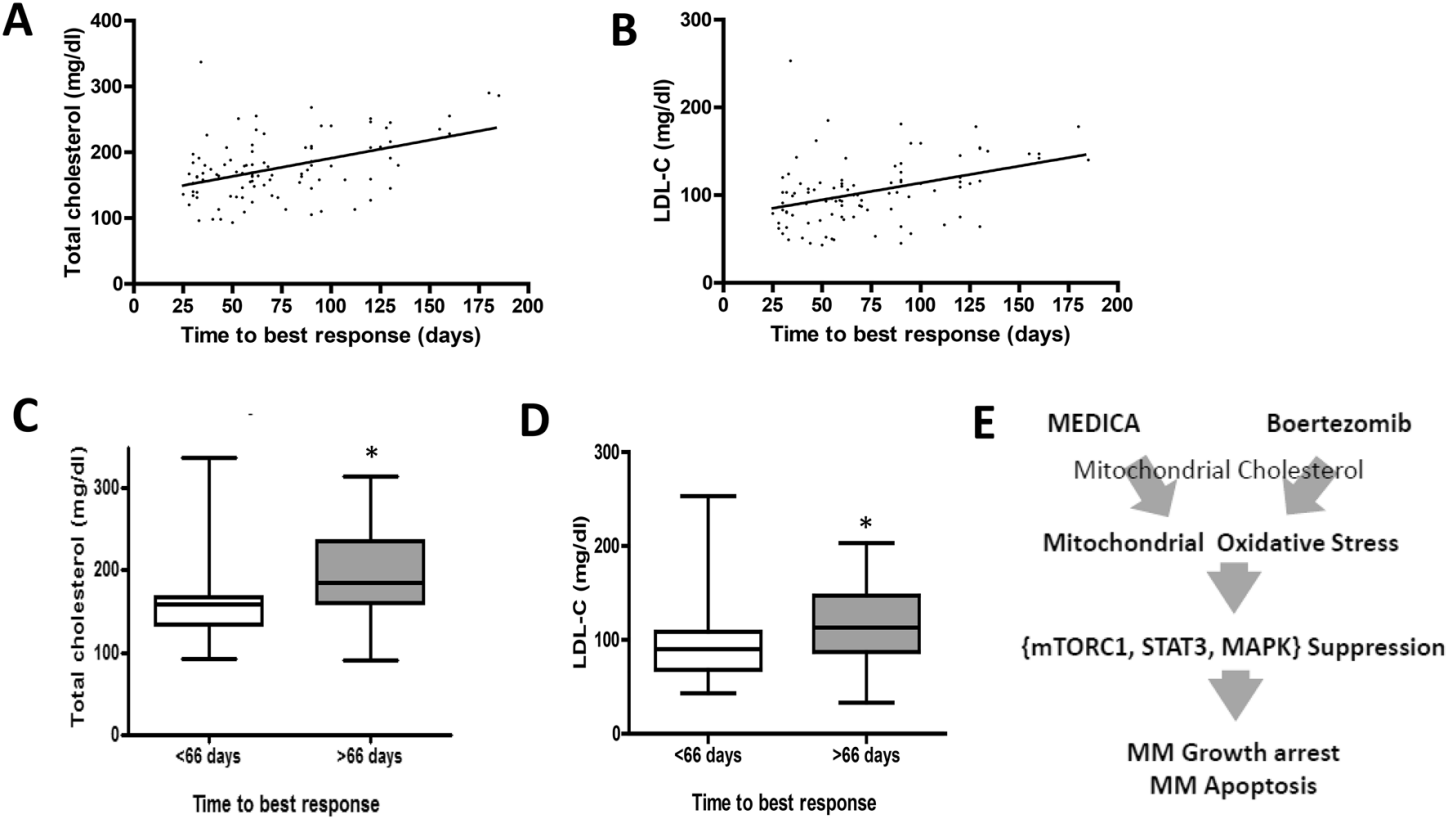
Response of MM patients to bortezomib in correlation to plasma cholesterol levels. **(A,B)** Plasma cholesterol (**A)** and LDL-C (**B)** levels of MM patients in correlation to respective time to best-response to bortezomib-based treatment protocol (n = 111). Pearson R = 0.44; R^2^ = 0.19 (p < 0.001) for **(A)**. Pearson R = 0.39; R^2^ = 0.15 (p < 0.001) for **(B)**. (**C,D)** Early (< 66 days) and delayed (> 66 days) response time of MM patients to bortezomib in correlation to their plasma cholesterol (**C)** and LDL-C (**D)** levels (n = 111)**.** *Significant as compared with time to best-response < 66 days (p < 0.005). (**E)** MEDICA and bortezomib converge onto shared targets.

The cholesterol/bortezomib interplay in MM cell survival was further verified by correlating the plasma cholesterol and/or LDL-cholesterol (LDL-C) levels of MM patients with their time to hematologic response to bortezomib-based protocol (“time-to-best-response”). A total of 111 MM patients were included. Of these, 13 (11.4%) patients were primarily resistant to bortezomib-based therapy and 98 (88.2%) had at least a partial response (PR), with a median time to best-response of 66.5 days (range 25–185). The time to hematologic response to bortezomib was found to be significantly correlated to the respective plasma cholesterol and LDL-C levels. Thus, the median time to best-response was shorter for patients having lower plasma cholesterol (Fig. 6A) or LDL-C cholesterol levels (Fig. 6B). In line with that, plasma cholesterol and LDL-C levels were lower in patients achieving best-response to bortezomib in less than 66 days as compared with late responders (Fig. 6C,D). These results were maintained upon setting a different median cutoff time of 100 days (Suppl Fig. 3A,B). In contrast to time to best-response, the overall response or the primary resistance to bortezomib therapy were not correlated with patient cholesterol levels (not shown). Also, in contrast to LDL-C, HDL-C levels were found to have no correlation with the time to best-response to bortezomib (not shown). Of note, 29 patients of the bortezomib-responsive cohort received statin therapy, but the correlation between the time to best-response to bortezomib and LDL-C levels was maintained upon excluding statin-treated patients (not shown).

## Discussion

MEDICA treatment is shown here to result in abrogating viability and inducing growth arrest and apoptosis of a variety of MM cells, including IL-6-constitutive (U266) and K-Ras-mutated (RPMI8226) MM cell lines, in the absence or presence of co-cultured BMSC, pointing to its anti-tumor effect even with stromal support. MEDICA efficacy was further verified in an MM xenograft animal model, resulting in decrease in tumor volume and mass, and in increased apoptosis. Suppression of MM survival by MEDICA adds to its reported efficacy in suppressing PyMT and HER2 breast cancer^23, 39^, and B-Raf(V600E) cancers^14^. Growth arrest and apoptosis of MM cells by MEDICA were accompanied by cell cycle arrest, decrease in cyclin-D, C-Myc, phospho-Rb, Mcl-1, Bcl-2 and Bcl-XL, and increase in cleaved caspase-3, PARP and annexin. Growth inhibition and apoptosis of MM cells by MEDICA were accounted for by mitochondrial oxidative stress, resulting in suppression of the STAT3, MAPK and mTORC1 transduction pathways. Suppression of the STAT3 and mTORC1 transduction pathways by MEDICA has previously been reported by us in a variety of cancer cells^14, 23, 39^, being accounted for by primary inhibition of mitochondrial complex I by MEDICA^39^. Suppression of MM survival by MEDICA was partially abrogated by counteracting ROS or by increasing mitochondrial cholesterol. Similarly, and in line with previous reports reviewed in ^5, 6^, suppression of MM cell survival by bortezomib is reported here to be accounted for by bortezomib-induced mitochondrial ROS, being abrogated by added anti-oxidants, or surprisingly, by increase in mitochondrial cholesterol. Indeed, suppression of MM cell survival by MEDICA was not additive with that of bortezomib, indicating that the two drugs may converge onto shared target(s) (Fig. 6E). Of note, bortezomib activity in inhibiting MM cell survival is primarily ascribed to proteasome inhibition, resulting in unfolded protein response, followed by endoplasmic^5^ and/or mitochondrial oxidative stress^6, 24–28^. In contrast to bortezomib, MEDICA efficacy is primarily due to its mitochondrial activity, while avoiding endoplasmic stress in MM cells (not shown). Hence, MEDICA may avoid side effects and resistance to proteasome inhibition.

In pursuing treatment modes for MM, high dose statins or bisphosphonates were previously reported to induce growth arrest and apoptosis of MM cells, being rescued by added mevalonate, geranyl-PP, farnesyl-PP or cholesterol^29–32^. Growth suppression by statins or bisphosphonates was ascribed to suppressing the farnesylation/geranylation/prenylation of small GTPases (e.g., Ras, RohA), or the disruption of lipid rafts due to cholesterol limitation^33^. Abrogation of MEDICA activity by added cholesterol as reported here is in line with the above previous reports. However, our findings further indicate that cholesterol may interfere with mitochondrial ROS production, and that cholesterol limitation may sensitize MM cells to selective cell apoptosis due to mitochondrial oxidative stress. This observation conforms to previous findings whereby mitochondrial cholesterol is reported to contribute to chemotherapy resistance in hepatocellular carcinoma^34^, while disruption of cholesterol uptake synergizes with chemotherapy in pancreatic adenocarcinoma^35^. The mode of-action of cholesterol in abrogating mitochondrial oxidative stress remains to be investigated.

The cholesterol/oxidative stress interplay appears to be of clinical relevance, as verified here by the time to best-response to bortezomib-based treatment protocols of MM patients. The time to best-response to bortezomib, but not the overall response rate, was found to be positively correlated with respective plasma LDL-C/cholesterol levels of MM patients. In addition, as most patients reported here were not treated with statins, the time to best-response to bortezomib in statin-treated patients may be ascribed to LDL-C lowering, rather than putative off-target activities of statins. Indeed, survival of myeloma cells is reported to be increased in the presence of LDL-C in the culture medium^36^. Of note, the clinical correlation reported here is based on a retrospective cohort and a univariate analysis of the data, thus requiring further prospective validation.

## Conclusions

Due to current standard-of-care, MM patients live for years with the disease under control. However, ultimately all patients do relapse at some point with 5-year survival rate of ~ 50%. Hence, MM is still an unmet need. Mitochondrial targeting by MEDICA is shown here to induce mitochondrial oxidative stress^40^ and to suppress MM oncogenic drivers, resulting in anti-tumor effect. MEDICA and bortezomib appear to share a common mode-of-action, and the efficacy of both may be enhanced by lowering plasma cholesterol. Hence, the combined activity of MEDICA, in inducing mitochondrial oxidative stress while lowering blood cholesterol, may translate into a novel effective treatment for MM patients.

## Supplementary Information

Supplementary Figures.

## Data Availability

All data generated or analysed during this study are included in this published article and its supplementary information files.

## Abbreviations

BMSC: Bone marrow stromal cells
CD-1: Cyclin D1
DCFDA: 2,7 Dichlorofluoresceine diacetate
FCS: Fetal calf serum
GSSG: Glutathione disulfide
LDL-C: LDL cholesterol
MM: Multiple myeloma
mPTP: Mitochondrial permeability transition pore
MTT: 3-(4,5-Dimethylthiazol-2-yl)-2,5 diphenyl tetrasodium bromide
PI: Propidium iodide
PR: Partial response
ROS: Reactive oxygen species
TMRM: Tetramethylrhodamine methylester perchlorate
