# Supplementary material for: Suppression of multiple myeloma by mitochondrial targeting
Source: Sci Rep. 2021 Mar 12;11:5862. doi: 10.1038/s41598-021-83829-2 (PMC7955047; doi:10.1038/s41598-021-83829-2)
Supplement: Supplementary file 1 — Supplementary Figures. [file 41598_2021_83829_MOESM1_ESM.pdf]

Supplementary File

Suppression of Multiple Myeloma by Mitochondrial Targeting

Eisen Y, Gatt ME, Hertz R, Smeir E, Bar-Tana J

Suppl FIG 1

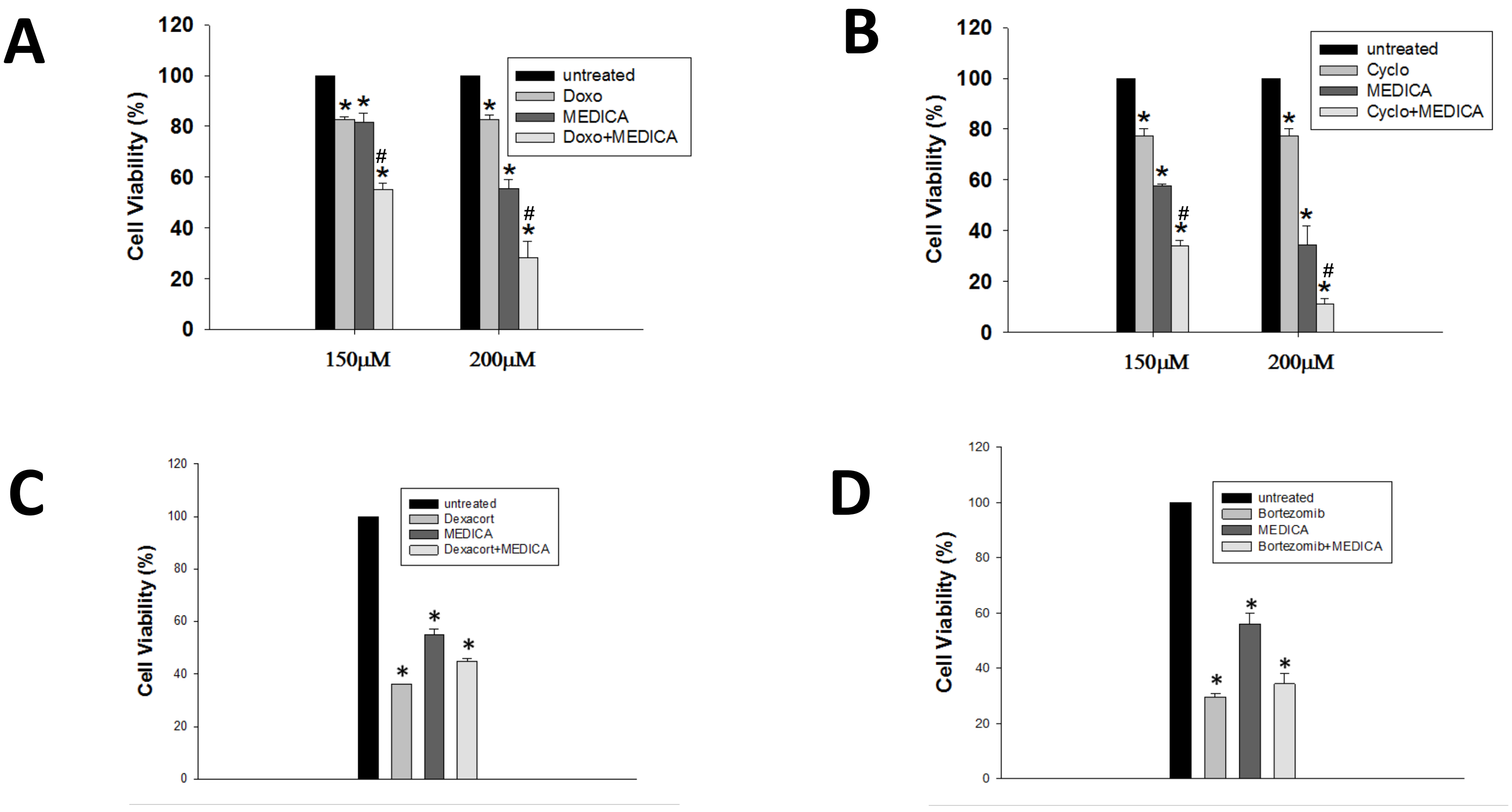
A
Cell Viability (%)
0
20
40
60
80
100
120
150µM
200µM
untreated
Doxo
MEDICA
Doxo+MEDICA
B
Cell Viability (%)
0
20
40
60
80
100
120
150µM
200µM
untreated
Cyclo
MEDICA
Cyclo+MEDICA
C
Cell Viability (%)
0
20
40
60
80
100
120
untreated
Dexacort
MEDICA
Dexacort+MEDICA
D
Cell Viability (%)
0
20
40
60
80
100
120
untreated
Bortezomib
MEDICA
Bortezomib+MEDICA

Suppl FIG 2

A

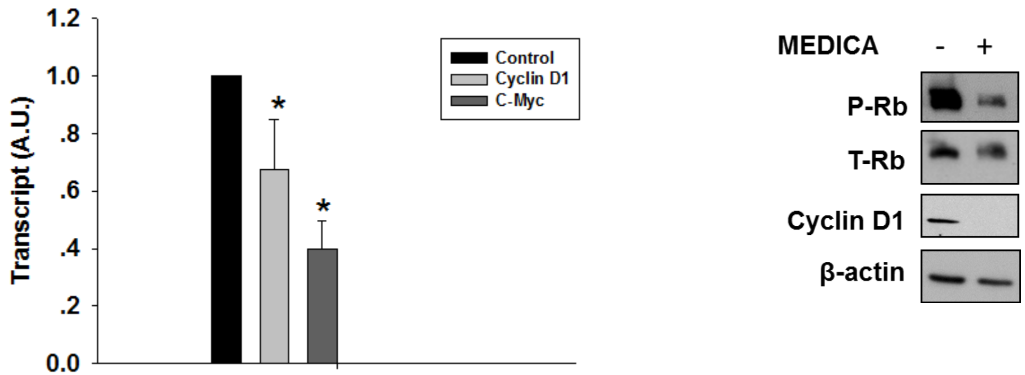

B

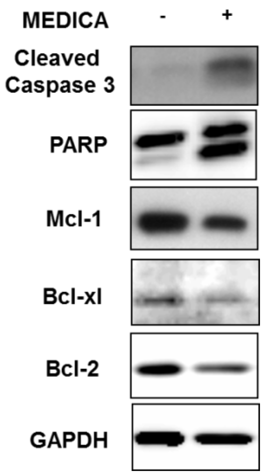

C

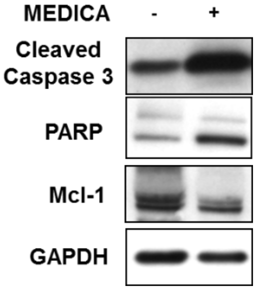

Suppl FIG 3

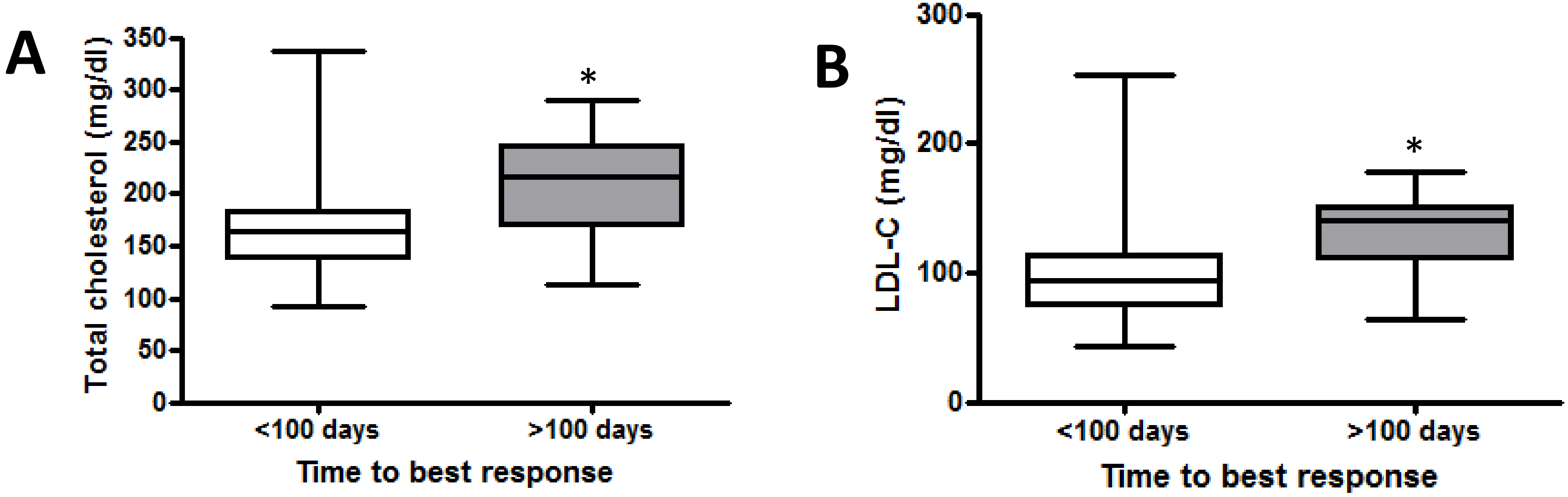
A
Total cholesterol (mg/dl)
350
300
250
200
150
100
50
0
*
<100 days
>100 days
Time to best response
B
LDL-C (mg/dl)
300
200
100
0
*
<100 days
>100 days
Time to best response

Supp Fig 4

Suppression of Multiple myeloma by Mitochondrial Targeting

Aisen Y, Gatt ME, Hertz R, Smeir E, Bar-Tana J

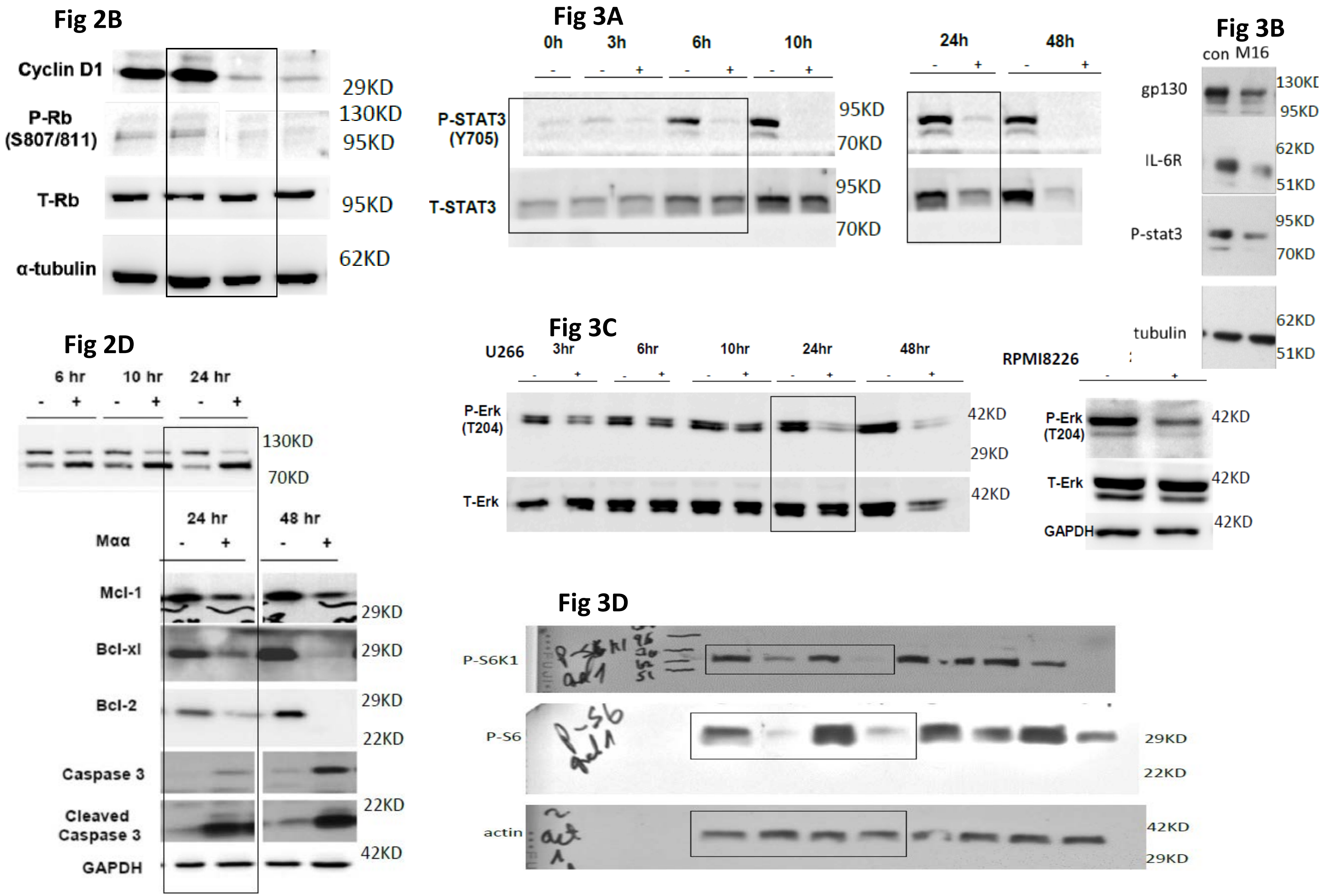

Supp Fig 5

Suppression of Multiple myeloma by Mitochondrial Targeting

Aisen Y, Gatt ME, Hertz R, Smeir E, Bar-Tana J

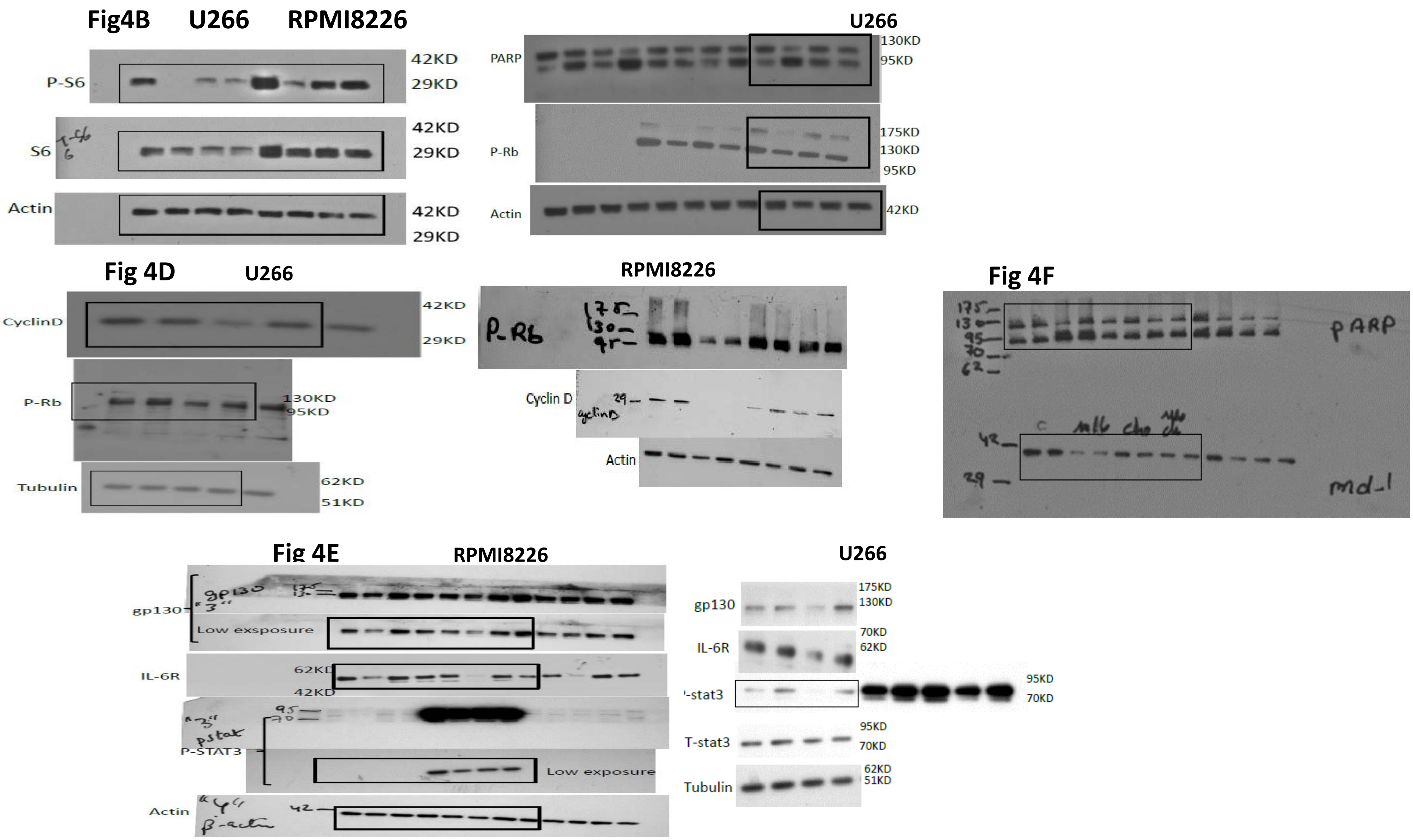

Supp Fig 6

Suppression of Multiple myeloma by Mitochondrial Targeting

Aisen Y, Gatt ME, Hertz R, Smeir E, Bar-Tana J

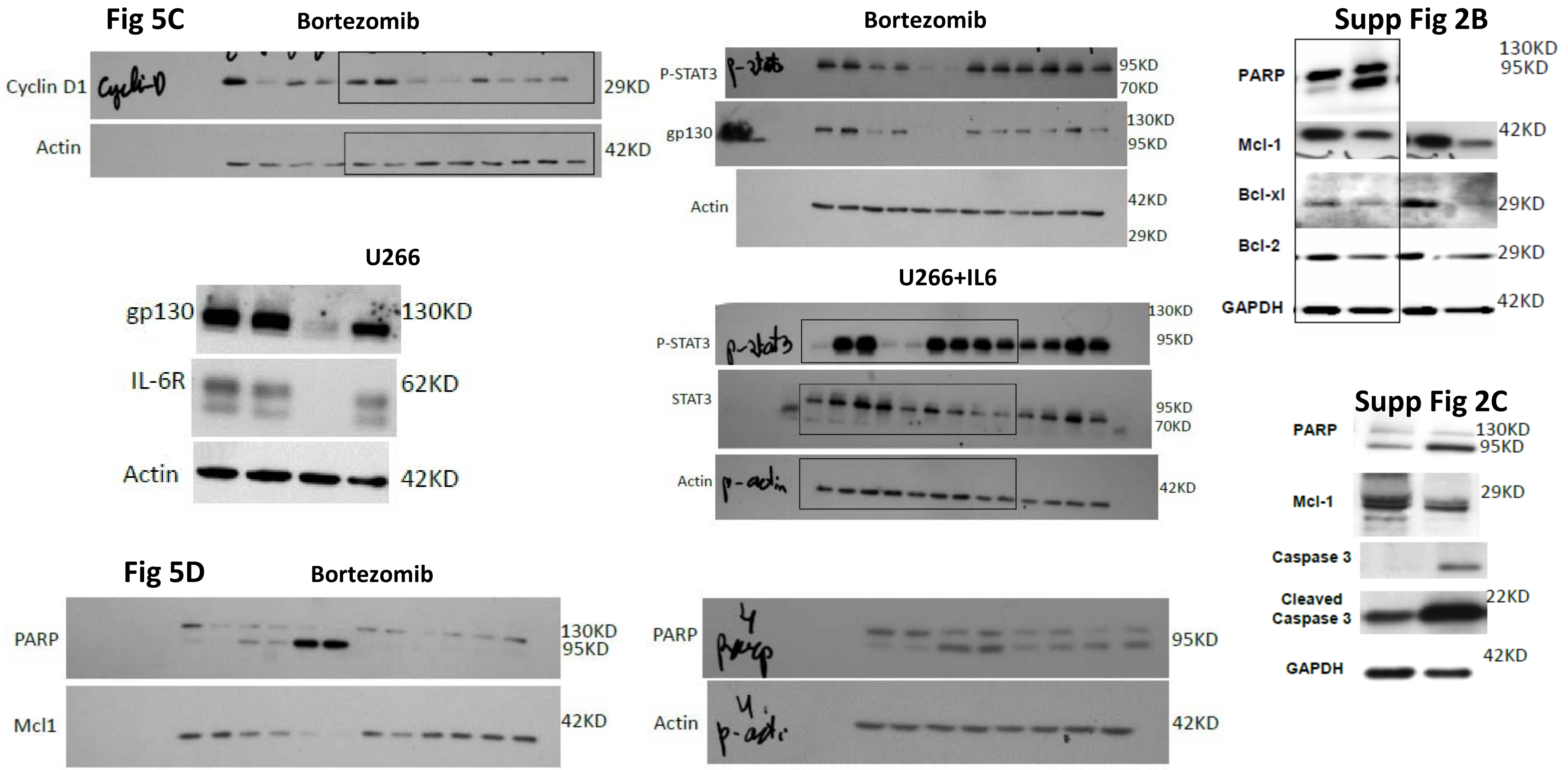

Legends to Supplementary Figures

Figure 1. MEDICA in combination with doxorubicin, cyclophosphamide, dexacort and bortezomib

RPMI8226 MM cells were treated for 48 h with 150-200 μM MEDICA and / or 400 nM doxorubicin (**A**), 2 mM cyclophosphamide (**B**), 1μM dexacort (**C**), or 5 nM bortezomib (**D**) as indicated. Mean ± SD of 5-8 replicates of 3 independent experiments. *Significant as compared with nontreated ($p<0.05$). #Significant as compared with the stand-alone drugs ($p<0.05$).

Figure 2. MM cell cycle arrest and apoptosis by MEDICA.

A. RPMI8226 MM cells were treated for 24 h with 200 μM MEDICA and 50 ng/ml IL-6. CD1 and C-Myc transcripts. Mean ± SD of 3 independent experiments. *Significant as compared with nontreated ($p<0.05$). Representative blots. **B.** RPMI8226 MM cells were treated for 24 h with 200 μM MEDICA. Representative blots. **C.** 5T33 mouse MM cells were treated for 24 h with 200 μM MEDICA. Representative blots.

Figure 3. Response of MM patients to bortezomib in correlation to plasma cholesterol levels.

Early (<100 days) and delayed (>100 days) response time of MM patients to bortezomib in correlation to respective plasma cholesterol (**A)** and LDL-C (**B)** levels**.** *Significant as compared with time to best-response < 66d ($p<0.001$).
